# Integrative Advances in Honey Bee Gut–Brain Interactions: From Microbiota Dynamics to Behavioral Regulation and Colony Health

**DOI:** 10.3390/insects17101017

**Published:** 2026-10-02

**Authors:** Ting Xiong, Zongwen Hu, Zhiyue Yang, Minghua Yang, Yahui Li

**Affiliations:** 1 Faculty of Animal Science and Technology, Yunnan Agricultural University, Kunming 650201, China; 2 Institute of Sericulture and Apiculture, Yunnan Academy of Agricultural Sciences, Mengzi 661101, China

**Keywords:** honey bee, gut–brain axis, gut microbiota, behavioral regulation, colony health

## Abstract

Honey bees provide a tractable model for studying the gut–brain axis in social insects. Unlike mammalian systems, the gut–brain axis in honey bees is jointly shaped by a relatively simple yet functionally specialized microbiota, physiological traits determined by caste differentiation and division of labor, and social interactions within the colony. Accumulating evidence indicates that gut microbes can influence neural function in honey bees through metabolic, immune, and neuroendocrine pathways, thereby affecting cognition, social behavior, immune homeostasis, and other host traits, and ultimately influences colony performance. Conversely, changes in host physiological state and environmental exposures can also reshape the gut microbiota, forming a dynamic feedback circuit between gut status and brain function. Given the high sociality of honey bees, microbial transmission and behavioral interactions among individuals may further amplify gut–brain axis effects and influence overall health of the colony. This review summarizes the establishment and temporal dynamic of the honey bee gut microbiome, the information transfer mechanisms between gut microbes and the host nervous system, and the effects of these interactions on key behaviors (learning, memory, foraging) and colony performance. It also examines major perturbing agents—pesticides, antibiotics, and pathogens—and explores strategies for colony health maintenance from the perspective of the gut–brain axis. Finally, the potential of microbiome-targeted interventions for improving colony health is critically evaluated. This review proposes an integrative framework from microbial dynamics to behavioral regulation and colony health, serving as a reference to promote basic research on the honey bee gut–brain axis and its applied translation for sustainable colony health management. These findings may also provide heuristic value for the study of human gut–brain interactions, neurobehavioral disorders related to microbial dysbiosis, and the development of microbiome-targeted health intervention strategies.

## Simple Summary

Honey bees are essential for crop pollination and ecosystem stability. Their gut microbes influence brain function, cognition, foraging, and social behavior, while environmental and social activities shape the gut microbiota. This review examines the mechanisms of the honey bee gut–brain axis and how pathogens and chemical stressors disrupt this balance, impairing colony health. Current research is limited by insufficient causal evidence, immature intervention strategies, and low ecological validity at the colony level. Future studies should move from descriptive associations to mechanistic validation, extend from individual physiology to colony ecology, and translate basic findings into practical applications. The honey bee gut–brain axis may also provide a valuable model for understanding human gut–brain interactions and neurobehavioral disorders associated with microbial dysbiosis.

## 1. Introduction

Honey bees are a quintessential social insect species characterized by strong resource sharing, highly precise division of labor, and a highly structured system of communication [1]. They represent keystone pollinator species and are widely employed as model systems for investigating the biology and evolution of social insects [2], playing an irreplaceable role in maintaining the stability of agroecosystems and safeguarding crop yields [3,4]. However, in recent years, global honey bee populations have been under sustained multiple pressures, including pathogen infection [5,6], pesticide exposure [7,8], nutritional imbalance [9,10], habitat degradation [11,12], and climatic fluctuations [13,14], all of which have affected their individual survival, foraging efficiency, social division of labor, and colony development to varying degrees. Honey bee health is no longer confined to a single pathogen or a single physiological process, but has instead manifested as a complex systemic syndrome co-determined by nutrition, immunity, neuroregulation, and behavioral output [15,16].

**Figure 1:**
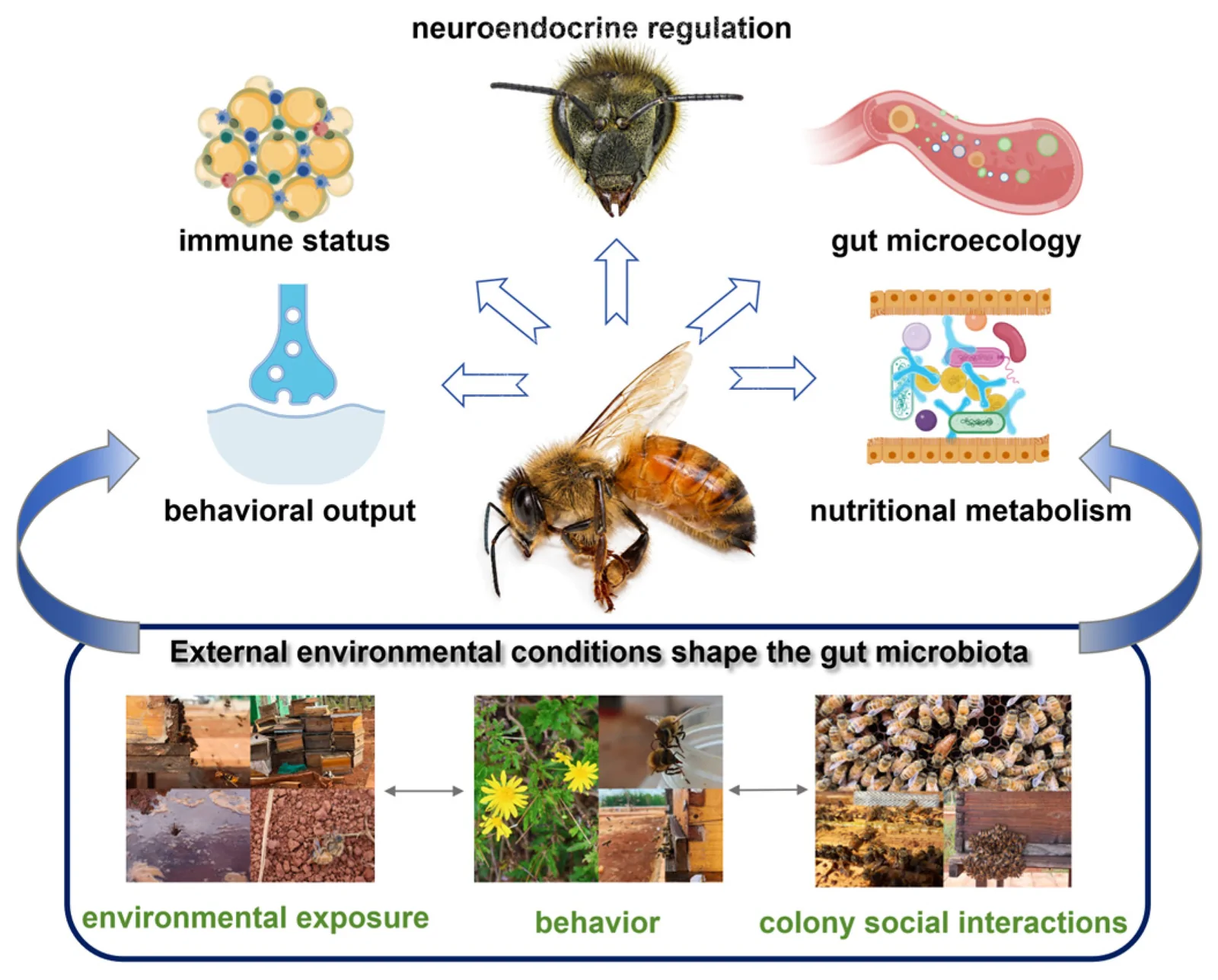
Multidirectional host–microbiota–environment feedback network in honey bees. This figure depicts the honey bee as a central host embedded in a dynamic feedback network involving the gut microbiota and external environmental conditions. Multidirectional interactions occur among the host (including nutritional metabolism, neuroendocrine regulation, immune status, and behavioral output), the gut microbiota, and the environment (including environmental exposure, nutritional resources, climate, and colony social interactions). The gut microbiota influences host physiology and behavior, while host physiology and behavior reciprocally shape the composition and stability of the gut microbiota. Through these interconnected feedback loops, the gut microbiota contributes to the regulation of learning and memory, foraging preferences, activity rhythms, and social behaviors in honey bees. Thin blue open arrows indicate the interactions between the honey bee host and immune status, neuroendocrine regulation, gut microbiota, nutritional metabolism, and behavioral output. The thick blue curved arrows on both sides indicate the regulatory effects of external environmental conditions on honey bee behavior, the gut microbiota, and related physiological processes. Graphic elements were provided by BioGDP.com and Ting Xiong, and the figure was assembled using Microsoft PowerPoint.

Against this background, gut microorganisms in honey bees have attracted considerable attention due to their crucial roles in nutrient metabolism, immune regulation, pathogen resistance, and host development [17,18]. With the rapid development of high-throughput sequencing technologies, bioinformatics and related fields, significant progress has been made in the application of microbiome research in honey bee science. Since then, a combination of sequencing and microbial culture methods has been employed to characterize the core members of the honey bee gut microbiota, their metabolic capabilities, and their roles in honey bee health. An increasing body of evidence indicates that gut microbes not only participate in digestion and nutrient conversion, but may also influence the neurophysiological processes and behavioral phenotypes of honey bees through metabolites, signaling molecules, and alterations in host immune status [19,20,21]. Particularly in social insects such as honey bees, microbial perturbation goes beyond physiological changes at the individual level; by affecting foraging [22], learning and memory [23], activity rhythms [24], and social behavior [25], it may further amplify into ecological consequences at the colony level. Consequently, honey bee gut microbiota research is transitioning from microecological description toward host functional regulation. With the convergence of microbiome research and neuroscience, the gut–brain axis has emerged as a research hotspot in life sciences in recent years. The concept of the gut–brain axis was first proposed by Professor Michael Gershon, a neurologist at Columbia University, who described it as the “second brain” of the human body, formed by the intestinal tract, the enteric nervous system, and gut microbiota [26]. In recent years, as the roles of gut microbes in host physiological regulation have been increasingly elucidated, the traditional concept of the gut–brain axis has been further expanded to encompass a complex communication network involving the gut microbiota. Given the vast microbial community residing in the intestinal tract, this bidirectional regulatory pathway—comprising the gut microbiota, the gut, and the brain—is commonly referred to as the microbiota–gut–brain axis [27,28]. From a mechanistic perspective, the gut–brain axis is not a single pathway but a regulatory system composed of multilayered signaling mechanisms. Gut microorganisms can modulate host energy metabolism, barrier function, and immune status by influencing the production of short-chain fatty acids [29], amino acid metabolites [30], and other bioactive compounds [31,32]. Meanwhile, gut-derived signals can also act on the central nervous system through neural transmission, endocrine regulation, and immune mediators, thereby influencing cognitive function, stress responses, and behavioral outputs [33,34,35,36]. Microorganisms are widely colonized in the insect digestive tract, where they can confer multiple benefits to their hosts, such as enhancing reproductive capacity [37,38] and lifespan [39], modulating developmental timing [40], improving the host’s ability to cope with environmental changes [41,42], and influencing behavioral performance [43,44]. In the honey bee system, the concept of the gut–brain axis should be understood as an integrated system constituted by microbial dynamics, host nutrient metabolism, neuroendocrine regulation, immune status, and behavioral feedback (Figure 1). Existing evidence suggests that gut microbes and their metabolites may influence neurotransmitter homeostasis, energy allocation, and stress responses in honey bees, thereby modulating learning and memory [45], foraging preferences [46], activity rhythms [24], and social behaviors [47]. Conversely, honey bee feeding behavior, environmental exposure, and colony contact patterns can also shape the colonization and stability of the gut microbiota [48,49,50]. Compared with mammals, honey bees lack vagus nerve–gut structure; however, their neuroendocrine, immune, and metabolic pathways are sufficient to form a functionally analogous framework for gut–brain interactions [51], making honey bees a valuable model for studying how microbes influence behavior and colony health. Despite advances in understanding the microbial composition, metabolic functions, and behavioral phenotypes of the honey bee gut microbiota, several critical issues remain unresolved in the field. First, most existing evidence focuses on correlations between microbiota changes and host phenotypes, whereas mechanistic studies that support causal inference are still relatively limited. Second, a systematic integration is still lacking regarding how gut microorganisms influence honey bee brain function through metabolites, immune mediators, or neuroendocrine pathways. Third, given the social nature of honey bees, it remains to be elucidated how dysregulation of the gut–brain axis at the individual level translates into colony-level health risks, aberrant division of labor, and diminished ecological adaptability.

Based on the above background, this review aims to focus on the recent advances in honey bee gut–brain interactions. Beginning with the composition and dynamic changes in the gut microbiota, we systematically summarize the potential mechanisms by which gut microbes participate in gut–brain signal transmission, with a particular focus on their effects on learning and memory, foraging behavior, social division of labor, and stress responses. Furthermore, we analyze the implications of these processes for honey bee health and colony homeostasis in the context of environmental stress. Finally, this review summarizes the challenges and knowledge gaps that remain in current research, and offers perspectives on future directions, including multi-omics integration, causal validation, and microbiome-based intervention. Through the above synthesis, we aim to provide a theoretical foundation for in-depth studies on the mechanisms underlying honey bee health maintenance, as well as for the development of relevant management and intervention strategies. Moreover, because honey bees possess a relatively simple and experimentally tractable gut microbiota, they may serve as a useful model for investigating general principles of microbiome–gut–brain interactions, with potential implications for other host systems.

## 2. Composition and Dynamics of the Honey Bee Gut Microbiota

### 2.1. Basic Composition of the Core Gut Microbiota in Honey Bees

The gut microbiota of honey bees exhibits a relatively simple composition with pronounced host specificity [52,53], which renders it a promising model system for studying host–microbe interactions. In contrast to the diverse and highly variable gut microbiota observed in mammals, the intestinal tract of adult worker honey bees harbors a limited set of core microbial taxa, with low overall diversity dominated by fewer than ten phylotypes, yet the functional roles of these taxa are relatively well-defined [54]. Ge et al. [55] defined core operational taxonomic units (OTUs) as those present at all five geographical sites and in at least 80% of individuals per site. They found that the gut bacterial communities of *Apis cerana* and *Apis mellifera* differed significantly, and that their core OTU sets also differed: 5 core OTUs in *A. cerana* versus 22 in *A. mellifera*, with only 2 shared. This indicates that within a single host species, core OTUs are highly prevalent, but across host species, the membership of the core OTU set can diverge substantially rather than being highly consistent. In addition, communities within the same species also differed significantly across geographical sites, especially over larger spatial or latitudinal gradients. In *A. mellifera*, Bridson et al. [55] found that nine literature-defined core phylotypes accounted for 98.9% of reads across 64 colonies in North-West England, and all detected core phylotypes were present in every colony; however, only 67% of individuals possessed all core phylotypes, and relative abundances and amplicon sequence variants (ASV) composition varied markedly. Therefore, the occurrence or high prevalence of core taxa is relatively conserved within a given host species, but the membership of core taxa, their relative abundances, and the overall community structure can vary with host species, geographical origin, colony genetic background, and environmental factors [55,56].

From a taxonomic perspective, the gut microbiota of worker bees is primarily composed of nine bacterial taxa. Among these, five taxa are detectable in nearly all honey bees and constitute the core gut microbiota: two Gram-negative taxa, *Snodgrassella alvi* (Betaproteobacteria) and *Gilliamella apicola* (Gammaproteobacteria) [57]; among Gram-positive microorganisms, *Bombilactobacillus* (formerly *Lactobacillus* Firm-4) and *Lactobacillus* (formerly *Lactobacillus* Firm-5) within the phylum Firmicutes are relatively abundant and widely distributed; *Bifidobacterium* (Actinobacteria), although present at relatively low abundance, is also commonly found in the guts of adult worker bees [58]. The remaining four taxa—*Frischella perrara* [59], *Bartonella apis* [60], *Bombella apis* [61], and *Commensalibacter* sp. (also referred to as Alpha 2.1) [52]—are less abundant and less stably distributed than the core members, yet they still occupy specific ecological niches within the honey bee gut. In addition, the crop and foregut harbor certain yeast-like fungi, environmentally acquired microorganisms, and transient colonizers, such as *Apilactobacillus*, *Bombella*, and *Fructobacillus* [62,63,64,65], as well as certain opportunistic pathogens (e.g., *Serratia* and *Hafnia*) [66]. Despite their low abundance, these microbes can influence honey bee survival and health. The presence and interactions of all these microorganisms collectively contribute to the maintenance of the gut microecological balance and stability within the host. It is worth noting that different studies may display minor discrepancies in nomenclature and taxonomic assignment, which largely stem from ongoing refinements to microbial phylogenetic frameworks and updates in molecular typing methods. Nevertheless, these discrepancies do not alter the fundamental fact that the honey bee gut microbiota is consistently dominated by a small set of core members. The “core” nature of the honey bee gut microbiota is reflected not only in taxonomic composition, but more importantly in its functional roles within ecological niches, exhibiting distinct spatial specificity across the complex digestive system of the host. The digestive tract of honey bees consists of the crop, midgut, and hindgut (comprising the ileum and rectum), with each region performing specific digestive and absorptive functions. The crop serves as a temporary organ for nectar storage in honey bees, and harbors a relatively low abundance of microorganisms, primarily those introduced via nectar or through contact with hives and beekeeping equipment, such as *Apilactobacillus* and *Bombella*. These microorganisms are typically associated with the initial processing of nectar [67]. The midgut, the largest digestive organ in honey bees, serves as a major site where the gut microbiota exert their functions. The honey bee midgut is a low-abundance, low-diversity, and dynamically fluctuating microbial habitat, *Gilliamella* can be detected in the midgut and may be involved in pollen degradation and toxic sugar metabolism [68]. The pylorus, serving as a transitional zone between the midgut and hindgut, harbors the non-core bacterium *Frischella*, which may perform specific functions during the transit of materials from the midgut to the hindgut [69]. In contrast, the hindgut represents the region with the densest microbial colonization. The hindgut of adult worker bees is lined with a stable cuticle, which helps maintain the relative stability of the internal gut environment [70]. Meanwhile, incompletely digested dietary compounds from the midgut accumulate in this region, providing abundant carbon and nitrogen sources for the gut microbiota and thus facilitating their growth and proliferation [71]. The ileum possesses well-developed internal folds that significantly increase the surface area available for nutrient absorption and stable microbial colonization [72]. Although the ileum is shorter in length than the midgut, it harbors substantially higher microbial abundance. The ileum is stably colonized by the two core taxa *S*. *alvi* and *G*. *apicola* [73,74]. *S*. *alvi* is generally considered one of the most representative members of the hindgut ileum, often forming biofilm-like structures attached to the gut wall, participating in the maintenance of the intestinal microenvironment, and providing a foundation for the colonization of other symbiotic bacteria [75]. *G. apicola* is closely associated with the degradation of pollen and plant-derived polysaccharides, and is capable of utilizing complex carbon sources, making it an important microbial member involved in the digestion of plant-based food in honey bees [76]. As the terminal segment of the hindgut, the rectum is nutritionally rich and attracts substantial microbial colonization, with dominant taxa including *L.* Firm-4, *L*. Firm-5, and *Bifidobacterium asteroides* [77]. Lactic acid bacteria and related taxa have been potentially involved in carbohydrate metabolism [78], lactate production [79], immune homeostasis, and the inhibition of certain pathogens [80], and may also participate in food fermentation and the transformation of metabolic intermediates. *B*. *asteroides* has been linked to carbohydrate utilization [76], hormone secretion [81], and task division [82] within the colony. These microbial taxa not only enrich the overall diversity of the gut microbiota but may also exert significant effects on honey bee intestinal health and overall physiological functions. Collectively, these members do not exist in isolation but rather form a relatively stable interaction network within the limited intestinal space, jointly contributing to the maintenance of honey bee gut homeostasis.

It should be emphasized that the gut microbiota of honey bees does not maintain a constant composition across all developmental stages. Although gut microbes can be vertically transmitted (e.g., *Snodgrassella* and *Gilliamella* can be transmitted ‘vertically’ from the mother colony to the young gynes [83]), perturbations of the microbiome during the larval stage do not affect the establishment of the core gut microbiota in adults. This is because the larval digestive tract environment, dietary sources, and social contact patterns differ markedly from those of adult bees, and the microbial load during the larval stage is typically low and unstable [84]. The core gut microbiota in the true sense is established gradually during the adult stage, particularly as worker bees acquire and transmit microorganisms after eclosion through interactions with nestmates [50], contact with feces [85], consumption of beebread, and participation in social food exchange [74]. This feature endows the honey bee microbiome with both individual host-associated and socially transmissible properties, making it more suitable than many solitary insects for studying host–microbe coevolution and the mechanisms of microecological stability at the colony level.

### 2.2. Colonization Patterns Across Different Developmental Stages

The establishment of the honey bee gut microbiota is not completed immediately after eclosion, but rather undergoes a gradual process of colonization and maturation. Newly hatched larvae are nearly devoid of bacteria [72], and microbial load remains extremely low in 3 to 4-day-old larvae; although a transient increase occurs by day 6, it declines markedly again during the pupal stage [86]. In terms of community composition, larvae almost completely lack the core microbial taxa characteristic of adult workers. In early-stage larvae of both Africanized and European honey bees, the gut is almost exclusively colonized by the Alpha 2.2 strain within the Acetobacteraceae family [87], whereas in *A. cerana* larvae, the few detectable bacteria are mainly from the phylum Firmicutes, with occasional traces of *L*. Firm-5 [88] or *G. apicola* [89] although substantial inter-individual variation exists. This low microbial load and instability are closely linked to the physiological developmental stage of the larvae. Developing larvae possess a discontinuous gut—their intestinal tract is not continuous, with no connection between the foregut and hindgut, and they do not void food residues until just before pupation [90]. Meanwhile, larvae feed exclusively on royal jelly secreted by nurse bees, which possesses potent antimicrobial activity and contains various peptide substances that inhibit Gram-positive bacteria, Gram-negative bacteria, fungi, and yeasts [91], thus making it unfavorable for extensive bacterial colonization. In addition, larvae lack the frequent social interactions and horizontal transmission routes that characterize adult bees—mechanisms through which the core gut microbiota of adult honey bees is primarily transmitted via social contact and fecal exposure, pathways that are largely absent during the larval stage. Therefore, the combination of multiple factors makes it difficult for the larval microbiome to form a stable and diverse community structure. More importantly, the microbial state during the larval stage does not determine the microbial composition in adulthood; rather, it serves as a starting point that is re-established after eclosion through social interactions among adult workers.

Subsequently, during the early developmental stages, worker bees acquire microbes through contact with pollen, honey, and adult secretions. As they age and their engagement in social behaviors increases, their gut microbiota gradually stabilizes, eventually forming the core microecological structure characteristic of the adult stage. This process demonstrates that the establishment of the honey bee gut microbiota is highly dependent on social transmission and behavioral contact rather than being determined solely by individual vertical inheritance. Allen et al. reported that the gut microbiota of worker bees is colonized within the first few days of adult life and matures into a relatively stable state within the first week [92]. Studies have shown that microbial colonization does not occur synchronously but follows a specific chronological order: approximately one day after eclosion, genera such as *Gilliamella*, *Frischella*, and *Snodgrassella* colonize first; by day 3 post-eclosion, genera including *Lactobacillus*, *Bifidobacterium*, and *Commensalibacter* also begin to colonize significantly [93]. This sequential pattern of colonization suggests a priority effect among different microbial taxa in the competition for ecological niches.

Worker bees of different ages and division of labor may also exhibit functional differences in gut microbial composition. Generally, young in-hive bees (nurses) have greater exposure to the colony environment and a relatively stable diet, resulting in less fluctuation in their gut microbiota. In contrast, older foragers are exposed to more complex external environments (e.g., diverse nectar sources, pesticide residues) and consequently show greater variability in their gut microbial communities. For example, studies have shown that foragers harbor a higher relative abundance of *B. asteroides* than in-hive nurses, and bees inoculated with this bacterium exhibited greater foraging intensity than those whose gut microbiota have been depleted. Conversely, the relative abundances of *Bombilactobacillus mellis* and *Lactobacillus melliventris* were lower in foragers compared with nurses [82]. These age-related microbial changes are likely closely linked to metabolic transitions [68], behavioral changes [94], and alterations in immune status [95] during the honey bee life cycle, and represent an important entry point for understanding the connections between the microbiota and gut–brain interactions.

### 2.3. Major Factors Influencing the Dynamic Changes in the Gut Microbiota

Although the honey bee gut microbiota exhibits core stability, its composition and abundance can still be significantly affected by a variety of internal and external factors. In honey bees, gut microbes not only participate in nutritional metabolism, immune homeostasis, and barrier maintenance but are also continually reshaped in response to changes in host age, developmental stage, dietary composition, social contacts, and environmental stressors. Therefore, understanding the major factors influencing the dynamic changes in the gut microbiota is fundamental to elucidating the mechanisms of brain–gut interactions and their roles in colony health.

Dietary structure is a fundamental determinant of microbial dynamics. Nectar serves as a primary source of carbohydrates for the colony, while pollen is rich in multiple nutrients, including carbohydrates, amino acids, lipids, and vitamins; together, they provide the essential nutrients required by the colony [96]. Although the polysaccharides in the pollen wall cannot be directly digested by honey bees, they provide important carbon sources for the gut microbial community [48,76]. Previous studies have shown that pollen consumption by honey bees significantly increases the abundance of total bacteria as well as core bacterial taxa in the bee hindgut. Conversely, pollen deprivation leads to a reduction in total bacterial load and core taxa in the rectum [97,98]. Pollen intake also stimulates the activity of glycoside hydrolases in the honey bee digestive tract, and the resulting metabolites—such as organic acids—contribute to host health [81,99]. Pollen type influences nutritional status, immune function, and gut microbial composition in honey bees [100], and pollen quality also affects the gut microbial community structure of developing workers [9]. Furthermore, nutritionally poor or monotonous food sources may destabilize the core gut microbiota and impair host metabolism and immune function. Previous studies have shown that diets consisting of pollen from a single floral species compromise overall honey bee health, particularly by reducing their capacity to resist pathogen infection [101,102].

Environmental stress represents a major driver of gut microbial fluctuations. Exposure to pesticides and insecticides, heavy metal contamination, climate change, habitat conditions, and migratory beekeeping [103,104,105] can all alter bee feeding behavior and physiological status, thereby perturbing the gut microecology either indirectly or directly. Certain sublethal concentrations of agrochemicals not only affect host neurobehavior but may also induce shifts in microbial composition and markedly reduce microbial diversity by compromising the intestinal barrier integrity, elevating oxidative stress levels, and altering immune status [106,107]. Such changes often involve more than a simple increase or decrease in individual taxa; instead, they reflect an overall decline in the stability of the entire microecological network.

Pathogen infection and parasitic pressure can profoundly reshape the gut microbial community [108]. Common honey bee pathogens, such as *Nosema* spp., and viruses (e.g., deformed wing virus, DWV, and chronic bee paralysis virus, CBPV) may indirectly modify microbial ecological niches by damaging intestinal tissues, depleting nutrient reserves, or inducing immune activation. Compared with healthy larvae, the gut bacterial load in honey bee larvae infected with European foulbrood or Sacbrood virus is significantly reduced [89]. Hubert et al. reported that high *Varroa destructor* infestation was associated with a decrease in the relative abundance of *B. apis* and an increase in the relative abundance of *S. alvi* in the gut microbiota of honey bees, along with changes in the balance of *Lactobacillus* communities [109]. The microbial dysbiosis caused by pathogens often further weakens host resistance, creating a vicious cycle of infection–dysbiosis–reinfection. This process is particularly important for understanding the protective role of the gut microbiota in honey bee health.

Host age [22,46], strain/lineage [50,110], and social behavior [82,94] also influence the stability of the microbial community. Owing to the highly social lifestyle of honey bees, the microbiota is not maintained in isolation within individual hosts but rather circulates and is redistributed across the colony network. Consequently, changes in the microbiota of individual bees often reflect shifts in colony-level ecology and social structure, rather than being merely localized phenomena within a single host.

## 3. Molecular and Physiological Mechanisms of the Gut–Brain Interactions in Honey Bees

At present, mechanistic evidence concerning gut–brain interactions in honey bees is uneven. Existing direct experimental studies mainly support local interactions between the gut microbiota and intestinal epithelial responses, the expression of immune-related genes, and susceptibility to intestinal pathogens. By contrast, many of the links between the gut microbiota and systemic metabolism, neuroendocrine signaling, and brain function are still based primarily on correlation analyses, inferences from microbial metabolic potential, or findings from other animal models. In this section, we classify the evidence into two categories. The first comprises direct evidence obtained in honey bees through the use of germ-free bees, mono-colonization, microbiota transplantation, or rescue experiments. The second comprises correlational evidence observed in honey bees, such as covariation between changes in the microbiota and metabolic, immune, or behavioral parameters.

### 3.1. Microbial Metabolism and Its Regulation of Host Nutritional Status

The honey bee gut microbiota first influences host physiological homeostasis by participating in the breakdown and transformation of nutrients [111]. The carbohydrates, proteins, lipids, vitamins, and minerals required for honey bee growth and development are derived primarily from nectar and pollen. The composition of these resources varies considerably according to their plant origin, and some components are not readily utilized by the host alone. Accordingly, gut microorganisms play an important role in substrate degradation and metabolism. For example, pectinases encoded by *G*. *apicola* and hemicellulases encoded by members of the genus *Bifidobacterium* can participate in the degradation of complex carbohydrates and plant-derived polysaccharides, generating metabolic intermediates that can be utilized by the host [76]. During polysaccharide hydrolysis, these microorganisms also release beneficial short-chain fatty acids (SCFAs) and absorbable monosaccharides, thereby promoting the development of the gut and the host as a whole [20,76,112,113]. Bottacini et al. [114] isolated the strain *B. asteroides* PRL2011 from honey bees and found that bifidobacteria in honey bees can perform respiratory metabolism under low-oxygen conditions and encode multiple enzymes to cope with toxic products generated during respiration. These functions may be important for host metabolism and physiology [112]. Collectively, these findings indicate that the honey bee gut microbiota has the potential to transform dietary substrates. However, they do not, by themselves, establish that the resulting metabolites are efficiently absorbed by the host or subsequently influence host behavior.

Previous studies have reported that honey bee gut microorganisms systemically affect the hemolymph metabolite profile [19], lipid metabolism [115], insulin signaling, and vitellogenin expression [20] through their metabolic products, thereby reshaping host energy allocation, nutritional status, and behavioral output. During the forager stage, high-intensity flight imposes greater demands on environmental exposure and energy homeostasis. At this stage, gut microorganisms may play a particularly important role in maintaining nutrient supply and energy balance by providing SCFAs as energy substrates, promoting lipogenesis, and regulating carbohydrate metabolism [68].

In addition, gut microorganisms may influence the intestinal environment and systemic metabolic homeostasis by producing organic acids [116], vitamins [49], and other small-molecule metabolites [117]. Although metabolic pathways in honey bees have not yet been characterized in a manner fully comparable to those of mammals, current evidence suggests that microbial metabolites can alter host nutrient sensing and physiological allocation, thereby providing a metabolic basis for changes in neural and behavioral functions [51]. In other words, the “gut” in honey bee gut–brain interactions is not merely a digestive organ, but also an important metabolic interface that bridges exogenous nutrition and central regulatory processes.

### 3.2. Interaction Between the Gut Microbiota and Immune Homeostasis

The gut microbial community can substantially influence honey bee health by modulating the immune system. However, for many ecologically and economically important insects, the relationship between the gut microbiota and immune function remains poorly understood. The honey bee intestinal epithelium constitutes an important barrier between the external environment and internal homeostasis. It is responsible not only for nutrient absorption but also for preventing pathogens from entering the host [17]. The presence of the gut microbiota generally helps maintain intestinal epithelial integrity and suppresses the proliferation of certain opportunistic pathogens through niche occupation, resource competition, and regulation of the local environment [118]. At the same time, the host immune system selectively restricts the excessive growth of certain microorganisms [119], thereby establishing a symbiotic equilibrium that is both stable and plastic.

In this process, gut microorganisms can shape the host’s basal immune state by inducing the expression of local immune-related genes and modulating antimicrobial peptide responses. In response to immune stimulation by Gram-negative and Gram-positive bacteria, four antimicrobial peptides (AMPs)—abaecin, apidaecin, defensin, and hymenoptaecin—are induced in the honey bee hemolymph [120,121]. Studies have found that, in the presence of gut bacteria, the expression levels of apidaecin, abaecin, and hymenoptaecin increase significantly, accompanied by activation of the c-Jun N-terminal kinase Pathway (JNK) signaling pathway. At the same time, the presence of gut bacteria can suppress the proliferation of *Nosema ceranae* [116]. Similarly, Kwong et al. [122] found that the honey bee gut microbiota can affect the hemolymph concentration of the AMP apidaecin, resulting in increased hymenoptaecin levels. *F. perrara* can colonize a specific region of the gut and induce morphological changes at the epithelial surface [69], which may activate host immune responses [117]. Existing evidence suggests an association between specific microbial communities and immune activation in the intestinal epithelium, indicating that microorganisms are not passive residents but can participate in setting and maintaining the host immune system [118]. In addition, higher activity of the *Bifidobacterium* community in the honey bee gut is associated with lower pathogen activity. This relationship suggests that *Bifidobacterium* may confer health benefits by modulating honey bee immune responses or excluding pathogens [123].

### 3.3. Role of Neuroendocrine Pathways in Gut–Brain Interactions

Honey bee gut microorganisms may influence behavior through the gut–brain axis. For example, bifidobacteria in the honey bee gut can induce the production of juvenile hormone, which may represent one mechanism of communication between the gut microbiota and the brain [72,81]. Honey bee behavior and physiological state are regulated by a range of hormones and neuromodulatory factors [19], including juvenile hormone, ecdysteroids, octopamine, dopamine, serotonin, and molecules involved in insulin-like signaling pathways. These signaling molecules are closely associated with energy allocation, age-related behavioral transitions, and the formation of learning and memory [119].

A substantial body of research has shown that gut microorganisms can produce neurotransmitters such as dopamine, serotonin, and γ-aminobutyric acid (GABA) [124,125,126]. In *Drosophila*, xylose isomerase (Xi) produced by *Lactobacillus brevis* negatively regulates octopaminergic signaling and thereby suppresses locomotor activity [127]. Jia et al. also demonstrated that the microbiota regulates aggressive behavior in *Drosophila* through positive modulation of the octopaminergic pathway [128]. In honey bees, juvenile hormone (JH), an important class of sesquiterpenoids, regulates insect development and behavior, including division of labor [129]. Kešnerová et al. [81] found elevated levels of JH III derivatives in the guts of colonized bees (CL) and this change was particularly associated with *B. asteroides*, suggesting that the gut microbiota may participate in regulating behavioral maturation in honey bees.

GABA is a major inhibitory neurotransmitter in the honey bee brain, and its receptors have been detected in multiple brain regions [130]. GABA is involved in the regulation of odor discrimination and memory formation [131,132]. The behavioral effects of orally administered GABA suggest that bacteria-derived GABA may act locally; dietary supplementation with GABA significantly reduces olfactory learning performance in honey bees [133]. GABA levels are elevated in the guts of honey bees mono-colonized with *B*. *asteroides* W8111 [50] or *Bombilactobacillus* W8089 [134]. Gut GABA levels are also higher in CL honey bees than in microbiota-free (MF) honey bees [20]. However, another study found no significant difference in gut GABA levels between CL honey bees or honey bees mono-colonized with different *B*. *asteroides* or *Bombilactobacillus* strains and MF honey bees [81]. Overall, current evidence supports GABA as an important candidate neurotransmitter signal in the honey bee gut–brain axis and suggests that gut microorganisms may produce or regulate GABA. Nevertheless, the complete causal chain—from microbial GABA production, to GABA entry into the brain, to changes in central GABA levels and subsequent behavioral regulation—remains inconsistent. These discrepancies may reflect strain-specific effects, microbial interactions, or differences in experimental design, and further research is required.

## 4. Effects of Gut Microbiota on Honey Bee Behavior

### 4.1. Effects on Learning and Memory

Learning and memory are among the most frequently tested phenotypes in honey bee behavioral research and also serve as important indicators for evaluating changes in neurological function. Honey bees possess a strong capacity for associative learning, enabling them to establish stable memories through olfactory, visual, and reward signals—a feature that has made them as a classic model in studies of neurobiology and behavioral ecology [135].

On the one hand, the stable presence of the core gut microbiota helps maintain the host’s nutritional status and metabolic balance, and these physiological foundations are important prerequisites for the normal functioning of neural functions. On the other hand, metabolites produced by gut microorganisms may alter the brain’s response thresholds to rewards and stimuli by influencing neurotransmitter homeostasis or modulating systemic signaling. An increasing number of studies have demonstrated that the gut microbiota can modulate sucrose sensitivity and olfactory learning and memory abilities in honey bees. Bees harboring a diverse gut microbiota display more typical gustatory responses to low-concentration sucrose solutions [20,134]. Notably, in 2024, a study reported that sucrose responsiveness in *A. mellifera* is not consistently influenced by the gut microbiota: neither bees experimentally inoculated with gut bacterial communities nor bees that naturally acquired the conventional gut microbiota showed significant changes in sucrose sensitivity [136]. This finding suggests that host physiological status and environmental factors need to be taken into consideration when evaluating the effects of the gut microbiota on host neural function and behavior. Compared with germ-free or antibiotic-treated bees, individuals colonized with a complete microbiota or specific strains exhibit higher learning efficiency [137].

The gut microbiota may regulate behavior by influencing brain function (Table 1). For example, colonization of honey bees with *G. apicola* increases the production of anandamide (AEA), a ligand in the endocannabinoid system, which in turn modifies honey bee learning and memory abilities. AEA can activate the insect-specific transient receptor potential channel AmHsTRPA in astrocytes, triggering calcium release [115]. Additionally, the gut microbiota can influence aversive learning and memory associated with defensive behavior in worker bees. Monocolonization of microbiota-free bees with the non-core gut bacteria *Enterococcus faecium* or *Enterococcus faecalis* restores aversive learning and memory, but this restoration is not accompanied by increased brain dopamine [45].

Current evidence regarding the influence of gut microbiota on honey bee cognition generally supports the view that the microbiota plays a supportive role in olfactory learning and memory in honey bees. Cabirol et al. [138] indicated that the cognitive enhancement effect is not conferred by a single bacterial genus but rather requires multiple core members of the intact gut microbiota; deletion of any single member or inoculation with only a single strain fails to reproduce the cognitive enhancement observed with the complete microbial community. This is complementary to the findings of Zhang et al. [137], who reported that under conditions of additional tryptophan (Trp) supplementation, monocolonization with *L*. Firm-5 (*L. apis* W8172) could promote memory by converting Trp into indole derivatives and activating the host aryl hydrocarbon receptor (AhR); however, without Trp supplementation, monocolonization alone was not sufficient to promote memory. Both studies used a similar experimental paradigm (germ-free bees + mono-colonization). Therefore, at this stage, we consider that there is a stable association between gut microbial status and honey bee learning and memory ability, and that under specific conditions it can significantly influence cognitive performance; however, the strength of its effects and the underlying mechanisms still need to be further validated using standardized experiments.

### 4.2. Effects on Foraging Behavior

Foraging behavior represents one of the most critical ecological behaviors in honey bees, involving multiple processes including floral resource detection, route memory, reward evaluation, transport decision-making, and feedback of information to the colony [139]. Gut microbiota may influence the initiation, duration, and choice preferences of foraging behavior by modulating energy metabolism and neural regulatory networks. For worker bees, foraging is not merely a simple outward collection activity but rather an integrated decision-making process closely linked to individual age, task allocation, colony demands, and environmental risks [140,141,142]. When gut microbes promote nutrient absorption and energy homeostasis, honey bees are more likely to maintain higher activity levels and stable foraging capacity. Conversely, if microbial dysbiosis leads to reduced metabolic efficiency, individuals may exhibit decreased activity, diminished flight endurance, or weakened responsiveness to floral stimuli. Using an automated behavioral tracking system under controlled co-housing conditions, Liberti et al. [22] found that the gut microbiota can advance the time of first arrival at the foraging arena by several hours in honey bees under laboratory conditions. In terms of foraging intensity, studies have revealed a causal relationship between specific microbial taxa and foraging behavior. Vernier et al. [46] observed consistent differences in gut microbial community structure between nurse bees and foragers. These differences were associated with variations in the abundance of individual microbes such as *Lactobacillus mellis*, *L. melliventris*, and *B. asteroides*.

Navigation ability constitutes another essential component of foraging behavior. Honey bees rely on spatial memory [143], view-guided navigation [144], and experiential accumulation [145] to accomplish their outbound and return flights, all of which impose high demands on both the nervous system and energy supply. The effects of honey bee gut microbes on navigation are predominantly indirect, primarily through their influence on metabolites that impact learning and memory [138] and modulation of taste sensitivity [20], thereby shaping foraging decisions and performance rather than by directly participating in path computation or spatial localization. Currently, research on the direct effects of gut microbes on honey bee navigation abilities, such as landmark memory or homing ability, remains limited and represents a promising direction for in-depth exploration.

### 4.3. Effects on Social Behavior

A hallmark feature of honey bees is their highly organized social behavior, in which individual behavior is not determined in isolation but is co-regulated by colony demands and age-related task transitions. Worker bees typically undergo a behavioral transition from in-hive tasks such as brood care, cleaning, and nest building to outdoor foraging. This age-related division of labor is governed by hormonal regulation, neurological status, and social environmental feedback.

Studies have found that the microbiota increases both the frequency and specificity of head-to-head interactions among honey bees, and also influences brain transcriptional processes related to amino acid metabolism and epigenetic modifications, thereby modulating the social network of honey bee colonies. It has been suggested that changes in chromatin accessibility and amino acid biosynthesis may serve as the underlying molecular mechanisms [25]. Additionally, the microbiota may regulate social behavior and social network structure by influencing brain amino acid metabolism and chromatin accessibility [25]. Through comparative analysis of the gut microbiota between hygiene-performing and non-hygiene-performing worker bees, researchers found that the relative abundances of three bacterial species—*Apilactobacillus kunkeei*, *B. apis*, and *F. perrara*—were significantly higher in hygiene-performing workers than in non-hygiene-performing workers. *Apibacter mensalis* was present exclusively in hygiene-performing workers and completely absent in non-hygiene-performing workers [47]. Furthermore, social behavior itself may reciprocally shape the gut microbiota. Through trophallaxis, body contact, and cohabitation within the nest, honey bees form a highly dense social transmission network [146] that facilitates not only the spread of pathogens but also of commensal microorganisms. In other words, the relationship between social behavior and the gut microbiota is not unidirectional but rather constitutes a closed-loop system of mutual reinforcement and mutual constraint. Understanding this is crucial for explaining why individual behavior and microbial status often exhibit synchronized changes within the same colony.

## 5. Role of the Gut–Brain Axis in Regulating Honey Bee Health Under Environmental Stress

### 5.1. Environmental Stress Disrupts Gut Microbiota Homeostasis

In natural and agro-ecosystems, honey bees are continuously exposed to a variety of environmental stressors, including pesticides, pathogen infections, parasitic pressure, heavy metals, and microplastics. These stress factors often act in combination, affecting the homeostasis of the gut microecology in honey bees, which in turn perturbs host brain function through multiple pathways—including metabolic, immune, neurological, and endocrine routes—ultimately manifesting as behavioral abnormalities and declines in colony health. Therefore, the gut–brain axis may represent an important pathway linking environmental stress to colony-level outcomes, rather than merely an isolated physiological phenomenon. However, current evidence is largely correlative, and causal mediation remains to be established. In particular, extrapolating individual-level behavioral and physiological changes to colony fitness requires further validation.

Pesticides, particularly neonicotinoids, pyrethroids, and other agrochemicals commonly used in agricultural production, are considered one of the major risk sources for impaired honey bee health [147]. Beyond their direct neurotoxicity, pesticides may also indirectly affect brain function and overall physiological status by altering gut microbial community structure. Pesticide exposure can lead to a reduction in core gut taxa, dysbiosis of microbial community structure, and proliferation of certain opportunistic taxa [148,149]. These changes may in turn induce metabolic disorders in the host [150], elevate oxidative stress levels [151], and alter the expression patterns of immune-related genes [152].

Pathogen infection and parasitic pressure are not only direct causes of honey bee health decline, but also important external factors that trigger gut–brain axis disruption. A study by Lau et al. [153] found that following *N. ceranae* infection, specific core gut microbial taxa, including multiple ASVs (amplicon sequence variants) of *Gilliamella*, were positively correlated with infection status. Honey bees infected with a high load of DWV exhibited a significant reduction in the relative abundance of beneficial genera such as *Fructobacillus*, *Lactobacillus*, and *Apilactobacillus*, while the bee gut-associated bacterium *Bartonella* was relatively enriched [95].

The disruption of honey bee microbial homeostasis by antibiotics is primarily manifested as dramatic changes in community structure and composition of microbiota. Allen et al. [92], through a study on tylosin treatment, found that the microbiome of 1-day-old bees underwent significant alterations in both community size and composition following antibiotic treatment, and this effect persisted for more than two weeks after the last administration, failing to recover to control levels by day 21. Notably, key symbionts such as *Bifidobacterium* and *Bombilactobacillus* were severely depleted, resulting in a persistent state of dysbiosis. Similarly, tetracycline exposure led to a marked reduction in the abundance of core gut taxa such as *Snodgrassella* and *Gilliamella* in treated bees, accompanied by a corresponding increase in the levels of opportunistic microorganisms [154]. Research by Shi et al. [155] further demonstrated that oxytetracycline exposure during the larval stage exerts a significant impact on the colonization and developmental trajectory of the gut microbiota in adult bees. Collectively, these findings reveal a critical fact: the perturbation of the honey bee microbiome by antibiotics is not a transient community fluctuation, but rather a structural disruption of the highly specialized core symbiotic microbiota.

Heavy metals enter honey bees mainly via ingestion of contaminated nectar, pollen, and water. A study by Li et al. [156] on *Apis cerana cerana* demonstrated that cadmium exposure altered the structural characteristics of both bacterial and fungal communities in the gut, increasing bacterial diversity while decreasing fungal diversity. Studies have shown that early-life exposure to copper significantly reduces gut microbial diversity in young bees, accompanied by disruptions in metabolic pathways. Copper exposure not only exerts its own toxic effects but also produces synergistic negative effects when combined with the pesticide imidacloprid, causing damage to gut health far exceeding that of either agent alone [150]. This combined toxicity suggests that, under real-world environmental conditions, the additive effects of heavy metals and other pollutants may pose a more serious threat to the honey bee microecology.

Chronic ingestion of microplastics also constitutes a direct cause of gut dysbiosis in honey bees. Following exposure to polystyrene microplastics (PS-MPs), the abundance of key *Lactobacillus* species in the bee gut declines significantly, while the abundance of certain bee gut-associated bacteria such as *Bartonella* increases [157]. Further studies have revealed that combined exposure to PS-MPs and flupyradifurone produced additive effects, reducing bee survival rates and damaging midgut epithelial cells more severely than exposure to either substance alone [158]. Moreover, co-exposure to microplastics and glyphosate similarly exacerbated gut dysbiosis and impaired cognitive behaviors in honey bees (e.g., sucrose responsiveness) [159], suggesting that the gut–brain axis may be involved in mediating these effects.

### 5.2. The Microbiota Modulates Colony Health via the Gut–Brain Axis

The microbial–gut–brain axis in honey bees constitutes a bidirectional communication network linking the gut microecology with the central nervous system, playing a critical role in regulating foraging behavior, learning and memory, and immune defense. Environmental stressors such as pesticides, pathogens, heavy metals, and microplastics have been shown to disrupt gut microbial homeostasis, thereby impairing honey bee cognitive function and colony health through this pathway. For example, exposure to nitenpyram can directly induce gut dysbiosis in *A. cerana cerana*, accompanied by reduced appetite and body weight. Such pesticides also disrupt the neurotransmitter network and proteomic balance in the brain. Supplementation with the core gut symbiont *Gilliamella* sp. G0441 significantly alleviated the above symptoms and restored olfactory learning ability in honey bees [160], providing direct evidence that “gut microbiota health is key to resisting pesticide neurotoxicity.” Phthalates (PAEs) disrupt the gut microbial community structure of worker bees and alter the levels of neuroactive substances in the brain and midgut, thereby compromising gut–brain axis homeostasis of *A. cerana* and leading to neurochemical imbalances in the Glu/GABA-Gln pathway [161]. Additionally, the organophosphate insecticide fenitrothion and the herbicide glyphosate have also been shown to alter gut microbial composition and affect cognitive behaviors in bees [152,162], indicating that multiple pesticides may interfere with gut–brain communication through common pathways.

Pathogen infection can trigger a cascade of cognitive decline by disrupting gut barrier function. Studies have shown that the effects of *N. ceranae* infection on honey bee cognition are age-dependent: infected nurse bees exhibited superior olfactory learning and memory performance compared to controls, suggesting premature foraging onset; however, when reaching the forager age, infected bees displayed learning and memory deficits [163]. DWA type A infection is not only associated with impaired learning and premature foraging in honey bees, but also shifts the brain transcriptional profile toward a forager-like state, suggesting that the virus may accelerate individual functional abnormalities and ultimately drive colony decline by reshaping the brain’s behavioral maturation program [164]. Heavy metals and microplastics have been associated with behavioral abnormalities, cognitive decline, and impaired immune function in honey bees, and these effects often co-occur with gut microbial disruption [150,165]. For example, in a recent study, combined exposure to polystyrene microplastics and glyphosate increased bee mortality, induced midgut damage and microbial dysbiosis, impaired sucrose responsiveness, and downregulated brain serotonergic synapse-related genes that correlated with shifts in core gut bacteria such as *Snodgrassella* and *Lactobacillus*, suggesting a potential role of the gut–brain axis [159]. Importantly, this study did not attribute the observed cognitive impairment exclusively to microbiota-mediated effects; direct pollutant effects and other mechanisms remain possible. Thus, the relative contributions of microbiota-associated and direct mechanisms to pollutant-induced behavioral and cognitive changes remain to be determined. Whether these individual-level effects scale to colony-level outcomes also requires further investigation.

Honey bees are highly social insects, in which changes in individual health are often rapidly translated into colony-level consequences through social networks. The destabilization of gut–brain axis interactions under environmental stress may ultimately manifest at the colony level as reduced foraging performance, decreased brood care efficiency, disrupted division of labor, and lower overwintering survival rates. From the perspective of population ecology, the disturbances in energy metabolism and behavioral abnormalities induced by gut microbial dysbiosis may alter the colony’s responsiveness to resource fluctuations and pathogen invasion. For example, a reduction in forager numbers directly weakens food input, abnormal behavior of in-hive workers affects larval feeding and nest maintenance, and compromised immune status increases the risk of disease transmission [166,167]. The interplay of these issues may ultimately lead to reduced colony stability and even colony decline. Thus, the gut–brain interaction is not merely a physiological mechanism at the individual level, but also a critical hub linking environmental stress to colony fate.

## 6. Future Perspectives

Although recent years have witnessed considerable progress in research on the gut–brain axis in honey bees, the field as a whole remains in a transitional phase from correlative observations toward mechanistic elucidation. Most existing studies are largely based on correlational analyses, making it difficult to definitively distinguish whether changes in the microbiota are causes or consequences of health alterations, or whether both are coincidental phenomena driven by common external factors. Therefore, the establishment of germ-free or semi-germ-free honey bee models, along with mono-colonization and synthetic community reconstitution experiments, will help clarify the genuine roles of specific microbes in host physiological regulation. Secondly, the functional characterization of the gut microbiota remains limited. Although the honey bee gut microbial community is relatively simple in composition, different strains may exhibit considerable variation in metabolic capacity, colonization traits, and host effects. Description confined to the genus or species level alone is often insufficient to reveal specific functions. Future efforts should integrate metagenomics, transcriptomics, metabolomics, and cultivation-based isolation techniques to further pinpoint key strains and their functional pathways. Thirdly, the influence of host background and environmental variables still requires adequate control. Bee strain origin, geographical location, seasonal variation, nutritional conditions, pesticide exposure, and colony management practices can all significantly influence gut microbial composition and host phenotypes. Therefore, the comparability of results across different studies needs to be improved, and establishing a unified framework for experimental design and data analysis is highly necessary. At the application level, research on the honey bee gut microbiota holds considerable potential for microbial intervention. On the one hand, core strains with probiotic functions can be screened to develop microecological agents aimed at enhancing that enhance colony stress resistance, nutrient utilization efficiency, and immune defense capacity. On the other hand, synthetic microbial communities with complementary functions can be designed to improve intervention efficacy and community stability. In addition, nutrition-oriented interventions based on beebread, pollen substitutes, or syrup formulations may also indirectly improve host health by modulating gut microbial community structure. However, the promotion of microbial interventions still requires caution. Exogenous strains may not necessarily achieve long-term colonization, and responses may vary across different colonies and ecological conditions. Any practical application should be based on strain identification, functional validation, and field safety assessment, avoiding the oversimplified adoption of probiotic strategies from other animal models. Overall, research on the honey bee gut–brain interaction is transitioning from descriptive studies to mechanistic investigations, and is progressively demonstrating translational value. Future breakthroughs in multi-omics integration, causal validation, and ecological context analysis would not only deepen our understanding of the regulatory mechanisms underlying honey bee health but may also offer novel microecological intervention strategies for colony health management. More importantly, honey bees—as a model organism characterized by a relatively simplified gut community, well-defined behavioral division of labor, and highly developed sociality—provide an important reference for understanding the roles of the microbiome in health and disease across other animal systems.

## Figures and Tables

**Table 1:** Summary of evidence that gut microbiota influence learning and memory in honey bees.

Paper	Target Bacterium	Behavioral Outcome	Behavioral Paradigm	Limitations of the Evidence
[137]	*Lactobacillus apis* W8172 (Firm-5)	Memory performance improves with tryptophan (Trp) supplementation. Without Trp supplementation, mono-colonization alone is insufficient to promote memory.	Reward olfactory proboscis extension reflex (PER) learning and memory	The cognitive effect of this single strain is dependent on substrate availability; colonization with the bacterium alone cannot be assumed to enhance memory.
[115]	*Gilliamella apicola*	W8136 + Linoleic acid (LA) significantly enhances learning and memory in honey bees. Knockdown of AmHsTRPA abolishes the promoting effects of W8136 + LA.	Reward olfactory PER learning and memory	The findings suggest that this bacterium may affect neural function through lipid signaling. The causal roles of the specific metabolic steps require further validation.
[45]	*Enterococcus faecium*, *Enterococcus faecalis*	Both species restore aversive learning and memory associated with defensive behavior. This recovery is not accompanied by an increase in brain dopamine.	Aversive learning and memory: SER (sting extension reflex) olfactory conditioning	The behavioral restoration induced by *Enterococcus* is not entirely dependent on increased brain dopamine levels, and the precise neural mechanisms remain incompletely understood.
[138]	BeeCom: *Gilliamella*, *Snodgrassella*, *Bombilactobacillus*, *Lactobacillus*, *Bifidobacterium*	The complete community enhances cognition. Removing any single member or inoculating bees with a single strain fails to reproduce the effect of the complete community.	Sucrose responsiveness assay; olfactory conditioning of the PER	Under the conditions tested, cognitive enhancement depends on a multispecies community and its coordinated activity, rather than on a single strain

## Data Availability

No new data were created or analyzed in this study. Data sharing is not applicable to this article.
